# Short-term results of percutaneous closure of a patent foramen ovale guided by transoesophageal echocardiography in patients with cryptogenic stroke: a retrospective study

**DOI:** 10.1186/s13019-022-01845-3

**Published:** 2022-05-03

**Authors:** Yilong Guo, Zhensu Shi, Yin Zheng, Caichan Xie, Jiao Yi, Zelun Chen, Yue Shu, Dexing Zhou

**Affiliations:** 1 Medical School of Chinese PLA, Beijing, People’s Republic of China; 2 Department of Cardiovascular Surgery, The Second Affiliated Hospital of Hainan Medical University, 48th of Bai Shui Tang Road, Haikou, Hainan 570311 People’s Republic of China; 3 Department of Special Medical Services, Hainan Cancer Hospital, No. 6th Changbin West 4th Street, Haikou, Hainan 570300 People’s Republic of China

**Keywords:** Patent foramen ovale, Cryptogenic stroke, Ultrasound foaming test, Interventional therapy, Transoesophageal echocardiography

## Abstract

**Background:**

A patent foramen ovale (PFO) is a risk factor for cryptogenic stroke (CS), and interventional therapy for PFO can reduce the recurrence rate of CS. However, interventional therapies are primarily guided by X-ray imaging, and data on regular post-surgical follow-up with the transthoracic ultrasound foaming test (UFT) are rare. Thus, this study aimed to assess the short-term (12 months) results of PFO occlusion guided by transoesophageal echocardiography (TEE) and the results of regular UFTs.

**Methods:**

Clinical records, echocardiographic data, and UFT results of 75 patients who underwent interventional therapy for PFO and CS were retrospectively analysed. The patients were grouped according to their preoperative UFT results: group A (n = 21), small volume of right-to-left shunts; group B (n = 22), moderate volume of right-to-left shunts; and group C (n = 32), large volume of right-to-left shunts. All patients were treated with an Amplatzer occluder under TEE guidance. UFT follow-up was conducted regularly until 12 months after surgery.

**Results:**

No significant differences in preoperative data, length of hospital stay, or operative time were noted between the groups (p > 0.05). The length of the PFO and diameter of the occluder differed between the groups as follows: group A = group B < group C (p < 0.001). Notably, 1 patient in group C developed recurrent stroke 11 months postoperatively, and 2 patients in group C developed atrial arrhythmia, which improved after 3 months of antiarrhythmic treatment. However, 19 patients still had positive UFT results 12 months postoperatively. Furthermore, the positive UFT rate 12 months postoperatively differed between the groups as follows: group A = group B < group C (p < 0.05). A preoperative large-volume shunt was negatively associated with a negative UFT rate 12 months postoperatively (OR = 0.255, 95% CI: 0.104–0.625).

**Conclusions:**

In patients with PFO and CS, interventional therapy guided by TEE could lead to satisfactory short-term (12 months) outcomes. Although the positive UFT rate gradually decreased, some patients still had positive UFT results 12 months postoperatively. Preoperatively, a large volume of right-to-left shunts and a longer PFO were the two risk factors for positive UFT results postoperatively. Further studies are required to clarify the relationship between postoperative positive UFT results and stroke recurrence.

## Background

The incidence of ischaemic stroke in China has been increasing annually; despite extensive examination, the aetiology is still unclear in approximately 40% of patients with stroke, and this is referred to as cryptogenic stroke (CS) [1, 2]. Studies have shown that a patent foramen ovale (PFO) is an independent risk factor for CS, especially in patients < 60 years of age with an atrial septal aneurysm or a large volume of right-to-left shunts [3, 4]. Compared with pharmacological treatments, PFO interventional therapy can significantly reduce the recurrence rate of stroke [5, 6]; therefore, methods of diagnosis and interventional therapies for PFO have been rapidly developed. The transthoracic ultrasound foaming test (UFT) is an important imaging modality that significantly improves the detection rate of PFO [7]; however, most PFO interventional therapies are currently performed using radiography, which is associated with the risks of radiation exposure. Furthermore, follow-up data on postoperative transthoracic UFT are lacking [4, 6]. Hence, this study assessed the short-term (12 months) effects of PFO interventional therapy guided by transoesophageal echocardiography (TEE) and the results of regular transthoracic UFTs after surgery.

## Methods

### Patients

This retrospective study included data of 78 patients who underwent PFO interventional therapy from April 2019 to July 2020. The inclusion criteria were as follows: (1) a clear diagnosis of CS and PFO; (2) age < 60 years; (3) transthoracic UFT performed strictly according to requirements (before surgery and at 3, 6, 9, and 12 months postoperatively). The exclusion criteria were (1) severe organ dysfunction; (2) atrial fibrillation or atrial flutter diagnosed before surgery; (3) other heart diseases requiring simultaneous surgical treatment; (4) patients with giant left atrium (left atrium diameter > 50 mm). Of these 78 patients, 2 were excluded due to preoperative atrial fibrillation and 1 was excluded due to severe liver disease. Finally, 75 patients were included. This study was approved by the Hainan Medical University Clinic Institutional Review Board. Due to the retrospective design of this study, the need for obtaining patient informed consent was waived.

### Transthoracic UFT

First, activated normal saline was prepared using two 10-mL syringes: the first syringe was used to draw 8 mL of normal saline and the other contained 1 mL of air and 1 mL of the patient’s blood. The syringes were connected to a three-way valve, and their contents were rapidly injected back and forth to fully mix the blood, normal saline, and air to obtain 10 mL of activated normal saline [8, 9]. Following this, two syringes were prepared for each patient, each containing 10 mL of activated normal saline.

Second, while the patient was in the supine position, an apical four-chamber view was obtained using transthoracic echocardiography. One syringe of activated normal saline was rapidly injected through the cubital vein of the patient. If a microbubble entered the left atrium within 10 cardiac cycles, it was recognised as a positive result. Subsequently, the patients were asked to perform the Valsalva manoeuvre (thoracic pressure ≥ 40 mmHg) for more than 10 s. The other 10 mL of activated normal saline was rapidly injected through the cubital vein [10]. The number of microbubbles in the left atrium during three cardiac cycles was recorded, and the partial flow of a PFO was graded as follows: no shunt, no microbubbles in the left atrium; small, 1–10 microbubbles in the left atrium; moderate, 11–25 microbubbles in the left atrium; and large, > 25 microbubbles in the left atrium [8, 9].

Third, the patients were divided into three groups according to their transthoracic UFT results: group A, small-volume shunt group; group B, moderate-volume shunt group; and group C, large-volume shunt group.

### Surgical methods

All surgeries were performed under general anaesthesia and guided by TEE. The length of the PFO was measured using TEE in the double vena cava section of the middle oesophagus, and an occluder was selected based on the measured PFO length. The right femoral vein was punctured, and the interventional track was established: right femoral vein—right atrium—PFO—left atrium. An Amplatzer occluder (Abbott Medical, Nathan Lane North Plymouth, MN, USA) was implanted along this track. Before releasing the occluder, the push–pull test was conducted to test the stability of the occluder, and the relationship between the occluder edge and cardiac structure was verified by TEE. After the occluder was released, the interventional catheter was removed, and pressure was applied over the right femoral vein puncture site to secure haemostasis. Finally, the patient returned to the general ward. Echocardiography was performed immediately and 12 h and 24 h after operation. If pericardial tamponade occurred, it was handled in time. Antibiotics were used to prevent infection from 30 min before operation to 24 h after operation. All the surgeries were performed by a single experienced surgeon.

### Follow-up

All patients were regularly treated with aspirin for 6 months (3–5 mg/kg/day). At 3, 6, 9, and 12 months postoperatively, all the patients returned to the hospital for transthoracic echocardiography, transthoracic UFT, chest radiography, and electrocardiography. All echocardiography and UFT results were evaluated by two doctors independently. If the reports of these doctors were inconsistent, the opinion of a third doctor was requested, and the opinion of the majority was the final data included in the analysis.

### Data collection and processing

Preoperative data included the sex ratio; age; weight, height; comorbidities; heart function; recurrence of stroke before surgery; sequelae of stroke before surgery; right ventricular (RV), left atrial (LA), main pulmonary artery (PA), pulmonary systolic pressures (PSP); pulmonary vascular resistance (PVR); left ventricular end-diastolic diameter (LVEDD); and left ventricular ejection fraction (LVEF).

Intraoperative data collected included the length of the PFO, operative time, and diameter of the occluder. Follow-up data collected included common postoperative complications (pericardial tamponade, occluder migration, and local vascular injury), length of hospital stay, stroke recurrence, atrial fibrillation, atrial flutter, and UFT results.

### Statistical analysis

Continuous data are expressed as means ± standard deviation (SD), while categorical variables are expressed as percentages. Between-group comparisons were performed using the one-way analysis of variance, chi-square test, or Fisher’s exact test, as appropriate.

Kaplan–Meier analysis was used to analyse the relationship between UFT results and follow-up time, and Cox regression analysis was used to analyse the effects of various factors on the postoperative UFT results. All statistical data were processed with SPSS version 19.0 (IBM SPSS, Armonk, NY, USA), and a *p*-value < 0.05 was considered reflective of the statistical significance.

## Results

### Preoperative information

Group A included 21 patients, with a mean age of 45.05 ± 8.992 years; group B included 22 patients, with a mean age of 45.09 ± 9.314 years; and group C included 32 patients, with a mean age of 41.25 ± 10.352 years. The youngest patient was a 27-year-old man in group C who developed hemiplegia after CS before surgery. There were no significant differences in the preoperative data between the groups. The clinical and echocardiographic characteristics of the study population are presented in Tables 1 and 2, respectively.

**Table 1:** Clinical characteristics of the study population

	Group A (n = 21)	Group B (n = 22)	Group C (n = 32)	*p* value
Female sex	14 (66.67%)	11 (50.00%)	12 (37.50%)	0.119
Age (mean ± SD, years)	45.05 ± 8.992	45.09 ± 9.314	41.25 ± 10.352	0.247
Weight (mean ± SD, kg)	60.92 ± 7.855	59.59 ± 11.548	56.72 ± 8.938	0.263
Height (mean ± SD, cm)	163.05 ± 7.965	160.68 ± 7.656	160.06 ± 5.224	0.287
Diabetes	2 (9.52%)	4 (18.18%)	1 (3.13%)	0.178
Hypertension	7 (33.33%)	8 (36.36%)	7 (21.88%)	0.467
Hyperlipidaemia	1 (4.76%)	4 (18.18%)	1 (3.13%)	0.112
Heart function (NYHA)				0.462
I	8	4	7	
II	10	14	21	
III	3	4	4	
Recurrence stroke before surgery	3	3	4	0.982
Stroke sequelae before surgery	1	1	3	0.719

*SD* standard deviation, NYHA New York Heart Association

**Table 2:** Echocardiographic characteristics of the study population

	Group A (n = 21)	Group B (n = 22)	Group C (n = 32)	*p* value
RV (mm)	23.24 ± 2.879	22.05 ± 4.326	21.47 ± 4.333	0.290
LA (mm)	31.48 ± 5.437	29.45 ± 3.776	28.69 ± 5.515	0.147
PA (mm)	21.29 ± 3.036	20.32 ± 2.255	20.47 ± 3.233	0.500
LVEDD (mm)	43.81 ± 2.839	42.82 ± 3.187	42.72 ± 2.667	0.367
LVEF (%)	63.00 ± 6.116	65.50 ± 2.596	64.98 ± 5.097	0.205
PSP (mmHg)	22.62 ± 4.031	23.09 ± 3.531	24.44 ± 2.590	0.121
PVR (dyn s cm^−5^)	150.10 ± 26.474	164.55 ± 20.871	158.47 ± 24.397	0.149

*RV* right ventricular, *LA* left atrial, *PA* main pulmonary artery, *LVEDD* left ventricular end-diastolic diameter, *LVEF,* left ventricular ejection fraction, *PSP* pulmonary systolic pressure, *PVR* pulmonary vascular resistance

### Perioperative data

All patients were successfully treated with PFO interventional therapy, and there was no incidence of perioperative pericardial tamponade, occluder migration, cardiac rupture, or malignant ventricular arrhythmia. There were 2 patients with an atrial septal aneurysm in this study, one was in group B and the other was in group C. PFO occlusion was performed successfully for both of them. No significant difference was found in the operative time and length of stay between the groups (*p* > 0.05). The length of PFO differed between the groups as follows: group A = group B < group C (*p* < 0.001). Patients in group C had significantly larger occluders than those in the other two groups (*p* < 0.001). The perioperative data of the study population are presented in Table 3.

**Table 3:** Perioperative data of the study population

	Group A (n = 21)	Group (n = 22)	Group C (n = 32)	*p* value
Time (min)	44.71 ± 25.714	35.64 ± 20.984	38.50 ± 25.821	0.464
Length of PFO (mm)	9.03 ± 4.616^a^	10.55 ± 3.546^b^	14.021 ± 3.047^a,b^	< 0.001
Occluder (mm)	19.67 ± 3.055^a^	19.91 ± 3.191^b^	23.25 ± 3.800^a,b^	< 0.001
Oesophageal bleeding	2	3	3	0.866
Length of stay (days)	12.24 ± 6.480	13.50 ± 8.478	13.00 ± 8.658	0.875

Statistical comparisons: comparison between groups, ^a^*p* < 0.01; ^b^*p* < 0.05

*PFO* patent foramen ovale

### Follow-up data

All patients in this study were followed up regularly for 12 months. There was no new incidence of migraine, pericardial tamponade, occluder migration, occluder-related valve dysfunction, death, bleeding, or oesophageal perforation within 12 months postoperatively. Only one 38-year-old female patient in group C developed transient recurrent stroke 11 months postoperatively. She was discharged without any sequelae after 7 days of treatment; she still had a positive UFT result 12 months postoperatively. Arrhythmia was not observed in groups A. One patient in group B who experienced supraventricular tachycardia immediately after the operation. The patient’s sinus rhythm was recovered by increasing the depth of anaesthesia and properly supplementing the blood volume. Two patients in group C developed atrial arrhythmias (alternating atrial fibrillation and atrial flutter) 3 months postoperatively. They were treated with amiodarone (200 mg/day) and recovered after 3 months. Eight patients developed mild chest tightness or chest pain immediately after the operation. However, all of them recovered within 3 months after the operation.

**Fig. 1:**
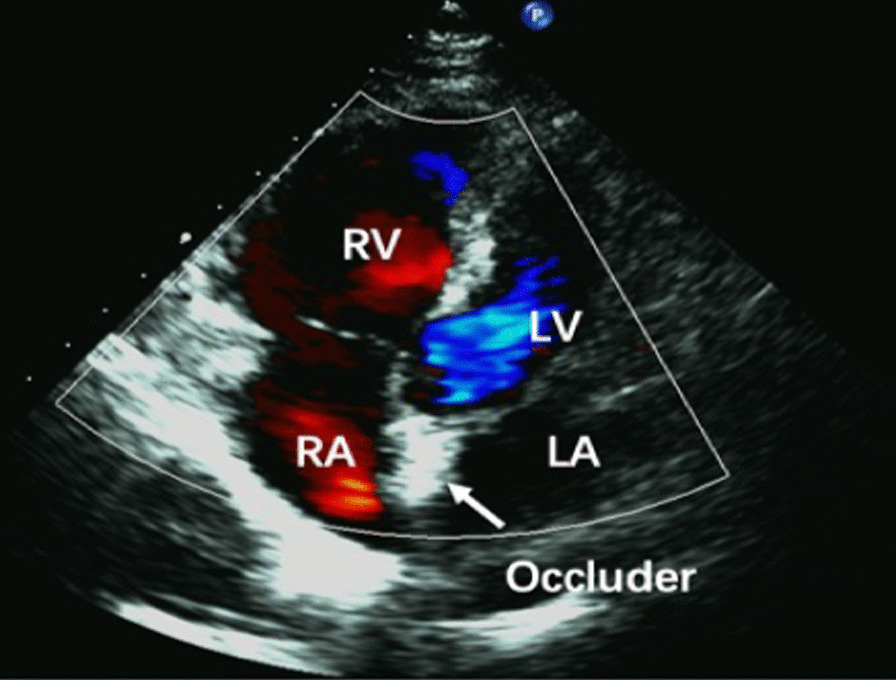
Transthoracic echocardiography shows blood flow after PFO occlusion. No residual shunt is noted between the LA and RA. *LV* left ventricular, *LA* left atrial, *RV* right ventricular, *RA* right atrial, *PFO* patent foramen ovale

**Fig. 2:**
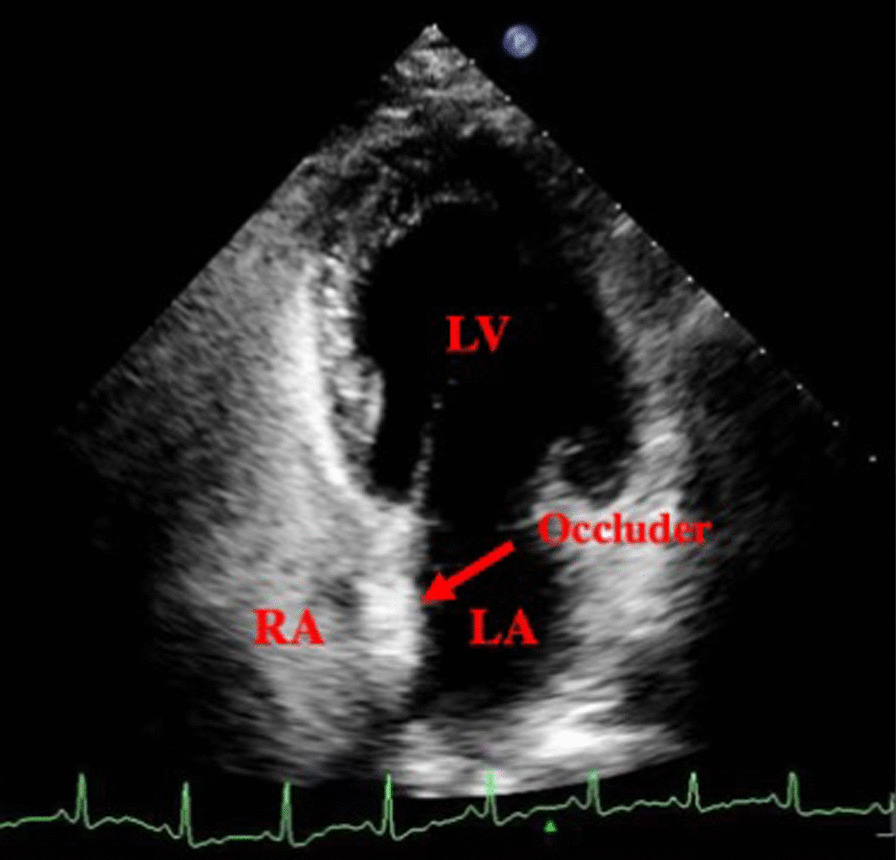
Negative transthoracic UFT result after PFO occlusion. There is no microbubble in the LA. *LV* left ventricular, *RA* right atrial, *LA* left atrial, *PFO* patent foramen ovale, *UFT* ultrasound foaming test

**Fig. 3:**
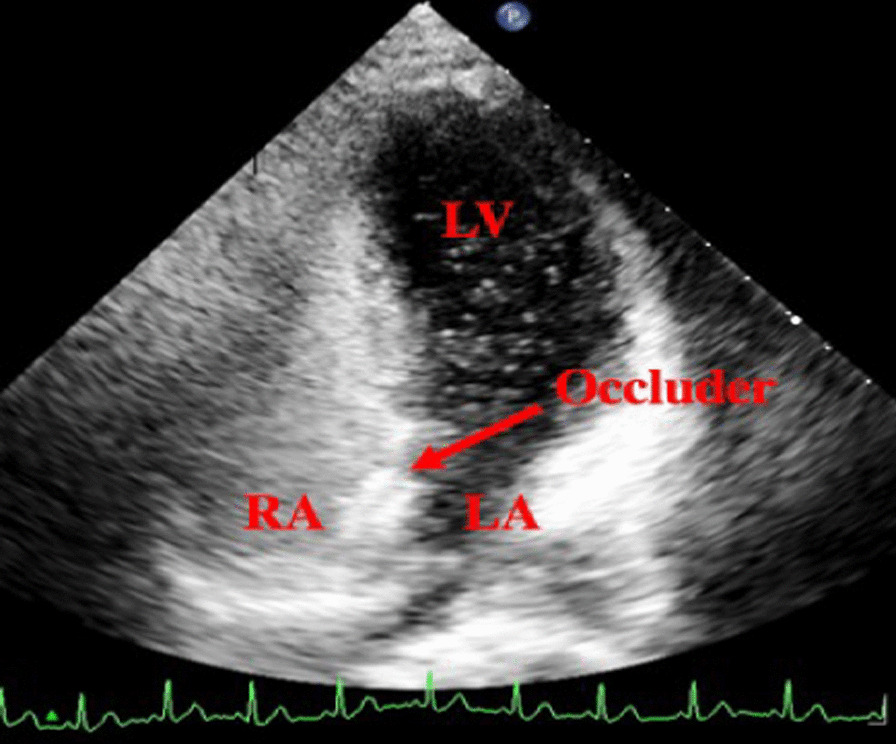
Positive transthoracic UFT result after PFO occlusion. There are more than 25 microbubbles in the LA and LV. *LV* left ventricular, *RA* right atrial, *LA* left atrial, *PFO* patent foramen ovale, *UFT* ultrasound foaming test

All patients underwent UFT regularly after surgery. The residual shunt on the atrial septum was excluded using transthoracic echocardiography before UFT (Fig. 1). Twelve months after surgery, no patients in group A had a positive UFT (Fig. 2). Notably, 3 and 16 patients in groups B and C, respectively, had positive UFT results (Fig. 3). The number of patients who had positive UFT results 12 months postoperatively differed between the groups as follows: group A = group B < group C (*p* < 0.05). The positive UFT results during follow-up are presented in Table 4.

**Table 4:** Positive results of UFT during follow-up

	Group A (n = 21)	Group B (n = 22)	Group C (n = 32)
Three months	17 (80.95%)	21 (95.45%)	32 (100%)
Six months	8 (38.10%)	13 (59.09%)	31 (96.88%)
Nine months	2 (9.52%)	7 (31.82%)	23 (71.88%)
Twelve months	0 (0)	3 (13.64%)	16 (50.00%)

*UFT* ultrasound foaming test

**Fig. 4:**
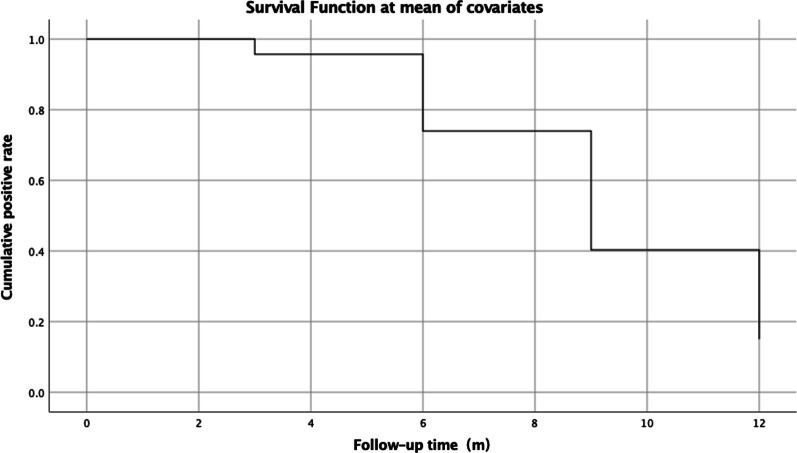
Life-table analysis: the cumulative positive UFT rate for all patients. *UFT* ultrasound foaming test

**Fig. 5:**
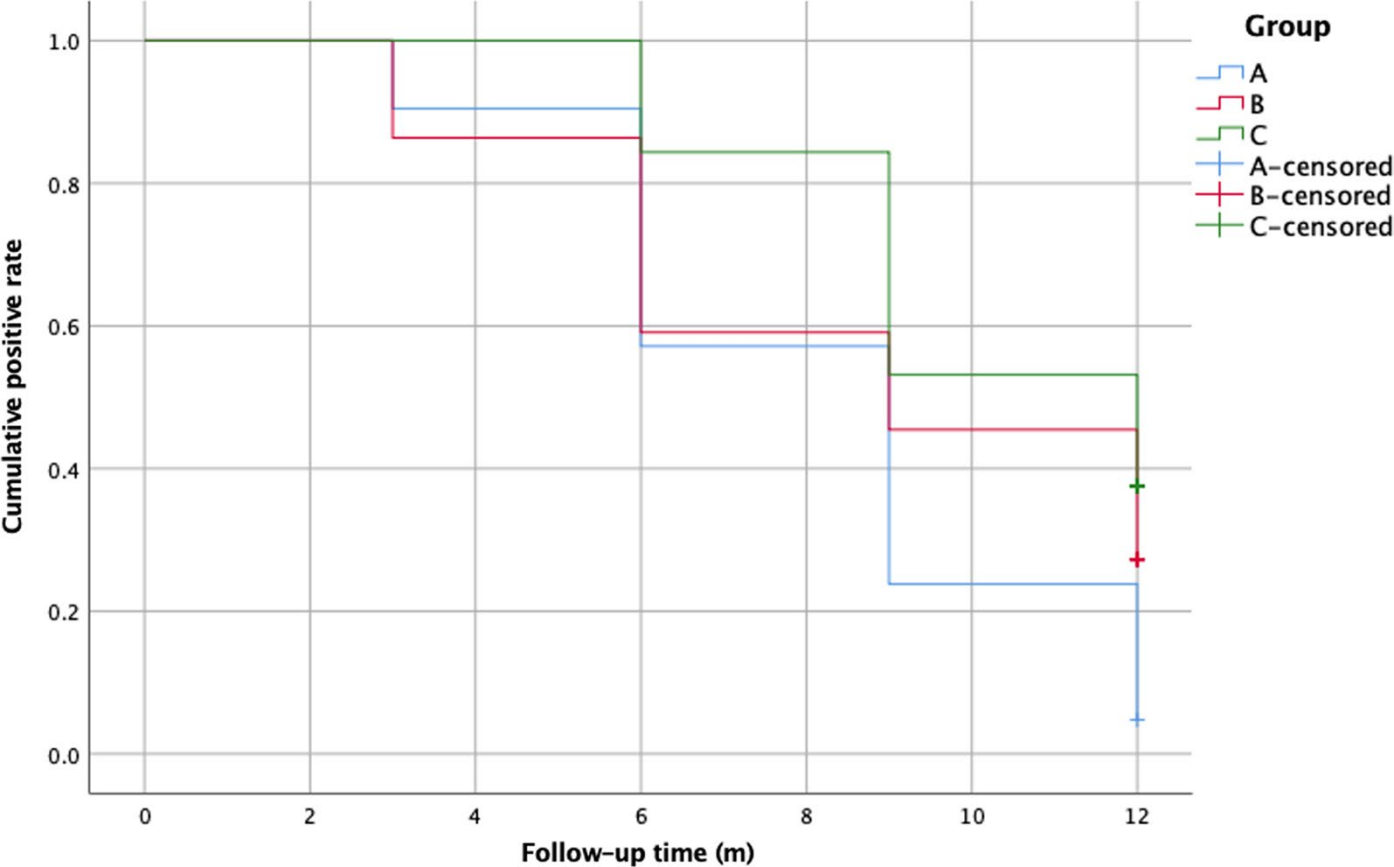
Cox regression analysis: the cumulative positive UFT rate for different groups. *UFT* ultrasound foaming test

Kaplan–Meier analysis showed that the positive UFT rate gradually decreased during follow-up (Fig. 4). Cox regression analysis revealed that the positive UFT rate in group C was higher than group A (*p* = 0.002) (Fig. 5). There was no significant difference in the positive UFT rate between groups A and B (*p* = 0.135). Moreover, a preoperative large-volume shunt was negatively associated with a negative UFT rate 12 months postoperatively (OR = 0.255, 95% CI: 0.104–0.625). Age, sex, weight, height, RV, PA, PSP, PVR, LVEDD, LVEF, and LA had no significant effect on postoperative UFT results (*p* > 0.05).

## Discussion

The foramen ovale is an important structure in the foetal heart that allows oxygenated blood to flow from the right atrium into the left atrium and ventricle, which then supplies blood and oxygen to the whole body [11]. After birth, with a decrease in the PA pressure and an increase in LA pressure, the foramen ovale closes spontaneously in approximately 75% of the population [12]. In others, it does not close, resulting in a PFO [13, 14]. Studies have shown that a PFO is a risk factor for CS; hence, studies on interventional therapy for PFO have been increasing for decades [15].

Initially, PFO interventional therapies were guided by X-ray imaging. However, its development was limited by the use of contrast agents and exposure to X-rays. Therefore, surgeons have recently begun to perform PFO interventional closure under the guidance of TEE [16, 17]. In our study, all patients were successfully treated with interventional therapy under TEE guidance, with no pericardial tamponade, death, or other major complications. The most important complications after PFO interventional therapy are recurrent stroke and atrial fibrillation [18, 19]; however, in this study, only 1 patient developed transient recurrent stroke 11 months postoperatively, and 2 patients developed atrial arrhythmias 3 months postoperatively. Therefore, these results suggest that PFO interventional therapy guided by TEE could lead to satisfactory short-term (12 months) results, which is consistent with the findings of previous studies [17].

Herein, the positive UFT rate gradually decreased. However, there were still 19 patients, most of whom belonged to group C, with positive UFT results at 12 months postoperatively; this was the most important finding of this study. Previous studies have shown that the recurrence rate of stroke after PFO occlusion in patients with CS is 2.0–5.0% [19], and the reason for this is still unclear. In our study, 1 patient in group C developed transient recurrent stroke 11 months postoperatively, and she still had a positive UFT result at 12 months postoperatively. A positive UFT result after surgery means that microthrombi and microbubbles can still be shunted from the right atrium to the left atrium through the PFO occluder. As the number of patients who developed recurrent stroke postoperatively in this study was small, further statistical analysis could not be conducted. Thus, we cannot rule out the possibility that a persistent positive UFT result after surgery is a risk factor for recurrent stroke. After detailed analysis, there were 2 possible reasons why 16 patients in group C still had positive UFT results 12 months postoperatively. First, the Amplatzer occluder, which is used worldwide, was used in this study. It has a metal-braided mesh structure without a film on the surface. Therefore, it cannot prevent the passage of microthrombi and microbubbles. However, the occluder is gradually embedded in the intima after it is released. This implies that when the occluder is completely embedded, microthrombi and microbubbles would be unable to pass through it, resulting in a negative UFT result [19, 20]. The present study shows that there are individual differences in the process by which the occluder is embedded. Moreover, patients with a large volume of right-to-left shunts before surgery will have a longer embedding process. Second, in this study, the length of the PFO and the diameter of the occluder in group C were larger than those in group A and group B, whereas the positive UFT rate 12 months postoperatively in group C was higher than that in groups A and B. Furthermore, the diameter of the occluder used in this study was equal to the length of the PFO + 10 mm. Considering all these findings, it could be concluded that with an increase in the PFO length, the diameter of the occluder increases and the embedding process is prolonged; hence, UFT positivity after surgery is observed in the long term.

This study has some limitations, as it was a retrospective, non-randomised study; therefore, some selection bias exists. Further prospective, randomised, large-scale, and long-term studies are required to clarify the changes in UFTs after PFO occlusion and to verify the relationship between postoperative positive UFT results and stroke recurrence.

## Conclusions

In patients with PFO and CS, interventional therapy guided by TEE could lead to satisfactory short-term (12 months) outcomes. Although the positive UFT rate gradually decreased, some patients still had positive UFT results at 12 months postoperatively. A large volume of right-to-left shunts and a longer PFO preoperatively were the risk factors for positive UFT results postoperatively. Further studies are required to clarify the relationship between postoperative positive UFT results and stroke recurrence.

## Data Availability

All data generated or analysed during this study are included in this published article.

## Abbreviations

PFO: Patent foramen ovale
CS: Cryptogenic stroke
UFT: Ultrasound foaming test
TEE: Transoesophageal echocardiography
RV: Right ventricular
LA: Left atrial
PA: Main pulmonary artery
PSP: Pulmonary systolic pressure
PVR: Pulmonary vascular resistance
LVEDD: Left ventricular end-diastolic diameter
LVEF: Left ventricular ejection fraction
SD: Standard deviation
